# Bioactive Secondary Metabolites of a Marine *Bacillus* sp. Inhibit Superoxide Generation and Elastase Release in Human Neutrophils by Blocking Formyl Peptide Receptor 1

**DOI:** 10.3390/molecules18066455

**Published:** 2013-06-03

**Authors:** Shun-Chin Yang, Chwan-Fwu Lin, Wen-Yi Chang, Jimmy Kuo, Yin-Ting Huang, Pei-Jen Chung, Tsong-Long Hwang

**Affiliations:** 1 Department of Anesthesiology, Taipei Veterans General Hospital, Taipei 112, Taiwan; E-Mail: nke0615@yahoo.com.tw; 2 Graduate Institute of Clinical Medical Sciences, College of Medicine, Chang Gung University, Taoyuan 333, Taiwan; 3 Graduate Institute of Natural Products, College of Medicine, Chang Gung University, Taoyuan 333, Taiwan; E-Mails: m9709109@stmail.cgu.edu.tw (W.-Y.C.); redbean1021@hotmail.com (Y.-T.H.); b9104208@stmail.cgu.edu.tw (P.-J.C.); 4 Department of Cosmetic Science, and Research Center for Industry of Human Ecology, Chang Gung University of Science and Technology, Taoyuan 333, Taiwan; E-Mail: cflin@gw.cgust.edu.tw; 5 National Museum of Marine Biology & Aquarium, Pingtung 944, Taiwan; E-Mail: jimmy@nmmba.gov.tw; 6 Chinese Herbal Medicine Research Team, Healthy Aging Research Center, Chang Gung University, Taoyuan 333, Taiwan

**Keywords:** elastase, formyl peptide receptor, *Bacillus* sp., neutrophil, superoxide

## Abstract

It is well known that overwhelming neutrophil activation is closely related to acute and chronic inflammatory injuries. Formyl peptide receptor 1 (FPR1) plays an important role in activation of neutrophils and may represent a potent therapeutic target in inflammatory diseases. In the present study, we demonstrated that IA-LBI07-1 (IA), an extract of bioactive secondary metabolites from a marine *Bacillus* sp., has anti-inflammatory effects in human neutrophils. IA significantly inhibited superoxide generation and elastase release in formyl-L-methionyl-L-leucyl-L-phenylalanine (FMLP)-activated neutrophils, but failed to suppress the cell responses activated by non-FPR1 agonists. IA did not alter superoxide production and elastase activity in cell-free systems. IA also attenuated the downstream signaling from FPR1, such as the Ca^2+^, MAP kinases and AKT pathways. In addition, IA inhibited the binding of *N*-formyl-Nle-Leu-Phe-Nle-Tyr-Lys-fluorescein, a fluorescent analogue of FMLP, to FPR1 in human neutrophils and FPR1-transfected HEK293 cells. Taken together, these results show that the anti-inflammatory effects of IA in human neutrophils are through the inhibition of FPR1. Also, our data suggest that IA may have therapeutic potential to decrease tissue damage induced by human neutrophils.

## 1. Introduction

Neutrophils play an important role in host defense systems against pathogen invasion and to clear damaged tissues [1]. However, there is growing evidence showing that overwhelming activation of neutrophils may become destructive and induce several inflammatory diseases, such as acute coronary syndrome, sepsis and acute respiratory distress syndrome [2,3]. For example, reactive oxygen species generated from activated neutrophils not only destroy pathogens, but also directly or indirectly damage surrounding tissues [4]. Therefore, the attenuation of neutrophil activation is a critical step to treat inflammatory diseases.

Formyl peptide receptor 1 (FPR1) is one of pattern recognition receptors which guide neutrophils to inflammatory sites and mediate downstream signaling pathways in the regulation of immune responses [5]. FPR1 can recognize bacterial or mitochondrial *N*-formyl peptides and has been shown to mediate either infectious inflammation or sterile inflammation [6,7]. Previous studies demonstrated that activation of FPR1 on neutrophils induces severe inflammatory response syndrome and organ damage [8,9]. Concerns have been suggested that the pharmacologic potential of FPR1 as a therapeutic target for the development of new drugs to treat inflammatory diseases [10].

There are increasing numbers of natural products in clinical development as therapeutic agents for the treatment of human diseases, especially from the marine ecosystem [11]. A number of secondary metabolites from marine organisms have been shown to display potential pharmacological activities, such as anti-inflammatory, bactericidal, and antiviral effects [12,13]. In the present study, our data suggest that IA-LBI07-1 (IA), an extract of bioactive secondary metabolites from a marine *Bacillus* sp., inhibits superoxide generation and elastase release in formyl-L-methionyl-L-leucyl-L-phenylalanine (FMLP)-activated human neutrophils through binding to FPR1.

## 2. Results and Discussion

### 2.1. IA Inhibits FMLP-Induced Superoxide Generation and Elastase Release in Human Neutrophils

**Figure 1:**
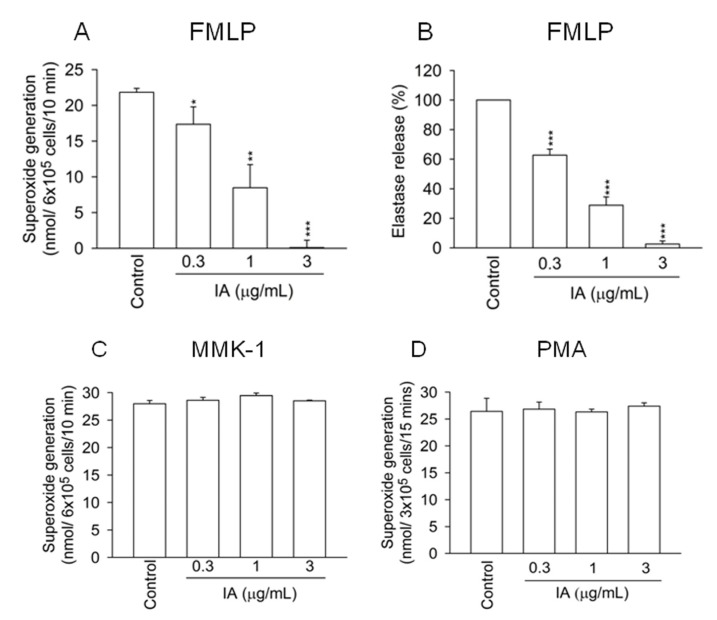
IA inhibits superoxide generation and elastase release in FMLP-activated human neutrophils. Neutrophils were incubated with IA (0.3–3 μg/mL) for 5 min. Superoxide generation (**A**) and elastase release (**B**) were induced by FMLP (30 nM) in the pretreatment of cytochalasin B (CB, 1 or 0.5 μg/mL). Superoxide generation was induced by (**C**) MMK-1 (100 nM) in the pretreatment of CB (1 μg/mL) or (**D**) PMA (5 nM). All data shown are means ± SEM. (n = 6 for A, n = 7 for B, n = 3 for C and D). *p < 0.05, **p < 0.01, ***p < 0.001 *versus* the control group.

There is growing evidence showing that overwhelming activation of neutrophils induces tissue destruction and organ dysfunction [14]. To investigate whether IA alters neutrophil functions, superoxide generation and elastase release from activated neutrophils were measured. Figure 1A shows that IA (0.3–3 μg/mL) had a dose dependent inhibitory effect on superoxide generation in FMLP (FPR1 agonist)-activated neutrophils. The 50% inhibitory concentration (IC_50_) of IA was 1.05 ± 0.29 μg/mL. Furthermore, IA also reduced elastase release in FMLP-activated neutrophils in a dose dependent manner with an IC_50_ value of 0.57 ± 0.09 μg/mL (Figure 1B).

In contrast, IA failed to alter neutrophil functions in Leu-Glu-Ser-Ile-Phe-Arg-Ser-Leu-Leu-Phe-Arg-Val-Met (MMK-1, FPR2 agonist)- and phorbol myristate acetate (PMA, protein kinase C activator)-activated neutrophils (Figure 1C,D). In addition, IA (0.3–3 μg/mL) did not cause the release of lactate dehydrogenase (LDH), suggesting that inhibition of the neutrophils’ respiratory burst and degranulation by IA was not attributable to cytotoxicity (data not shown). These data indicate that IA specifically inhibits respiratory burst and degranulation of FMLP-activated neutrophils.

### 2.2. IA Does not Show Inhibition in Cell-Free Systems

To determine whether IA directly scavenges free radicals and inhibits elastase activity, the inhibitory effects of IA were tested in cell-free systems. The superoxide and free radicals scavenging effects of IA were respectively assayed in the xanthine/xanthine oxidase system and DPPH assay. IA, at the concentration of up to 3 μg/mL, failed to reduce superoxide production and DPPH radicals in cell-free systems.

**Figure 2:**
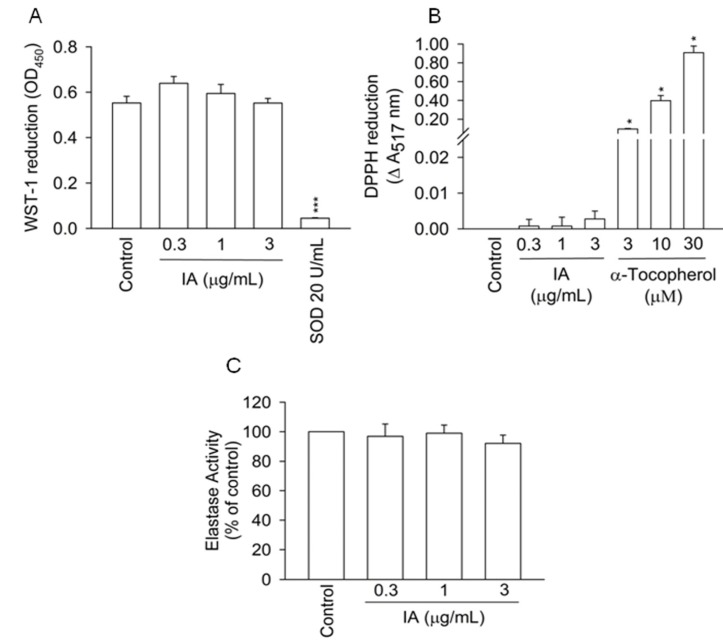
IA fails to inhibit free radicals production and elastase activity in cell-free systems. Antioxidant effects of IA were investigated in the cell-free xanthine/xanthine oxidase system and DPPH assay. Reduction of (**A**) WST-1 and (**B**) DPPH were measured at 450 and 517 nm, respectively. (**C**) The activity of extracellular elastase in the presence of IA was measured at 405 nm. All data shown are means ± SEM. (n = 7 for A, n = 4 for B and C). * p < 0.05, *** p < 0.001 *versus* the control group.

Superoxide dismutase (SOD) and α-tocopherol were used as the positive control, respectively (Figure 2A,B). We also found that IA had no direct inhibitory effect on elastase activity (Figure 2C). These results indicate that IA does not inhibit free radicals production and elastase activity in cell-free systems.

### 2.3. Protein Kinase A (PKA) Pathway Does not Mediate the Inhibitory Effects of IA

**Figure 3:**
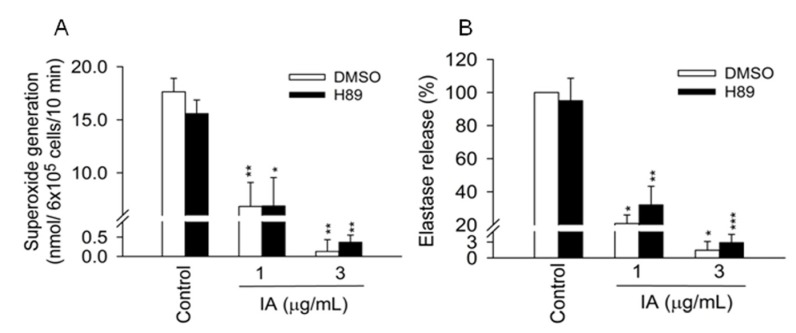
cAMP/PKA pathway is not involved in the inhibitory effects of IA. H89 (3 μM) was preincubated for 5 min before the addition of IA in human neutrophils.(**A**) Superoxide generation and (**B**) elastase release were induced by FMLP/CB. All data shown are means ± SEM. (n = 4 for A, and n = 6 for B). * *p* < 0.05, ** *p* < 0.01, *** *p* < 0.001 *versus* the control group.

Previous studies showed that an increase in intracellular cAMP levels and subsequent activation of PKA are associated with the inhibition of multiple intracellular activities, including the respiratory burst and the degranulation of neutrophils [15,16]. In the following experiments, we investigated whether the cAMP/PKA pathway is involved in the inhibitory effects of IA. H89 (3 μM), a PKA inhibitor, failed to reverse the inhibitory effects of IA on superoxide generation and elastase release in activated cells (Figure 3A,B).

### 2.4. IA Attenuates Ca2+ Mobilization Induced by FMLP

**Figure 4:**
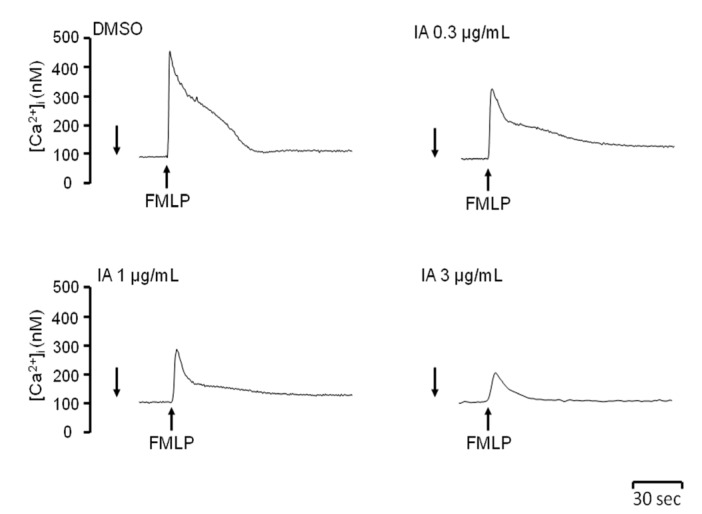
IA attenuates peak [Ca^2+^]_i_ induced by FMLP. Neutrophils were labeled with Fluo-3 acetomethoxyester (Fluo-3/AM, 2 μM) and then incubated with IA. Calcium mobilization was activated by the addition of FMLP (30 nM). The traces shown are from five independent experiments.

FMLP activates neutrophils by binding to the G protein coupled receptor (GPCR). Stimulation of GPCR induces transient elevation of intracellular Ca^2+^ concentration ([Ca^2+^]_i_) and subsequently activates many neutrophil functions [17]. To determinate whether IA alters Ca^2+^ signals in activated neutrophils, the peak [Ca^2+^]_i_ was assayed. IA had a dose-dependent inhibitory effect on the peak [Ca^2+^]_i_ in FMLP-activated neutrophils (Figure 4). These results suggest that IA attenuates Ca^2+^ signals in FMLP-activated neutrophils.

### 2.5. IA Inhibits Mitogen-Activated Protein (MAP) Kinases and AKT Phosphorylation in FMLP-Activated Neutrophils

**Figure 5:**
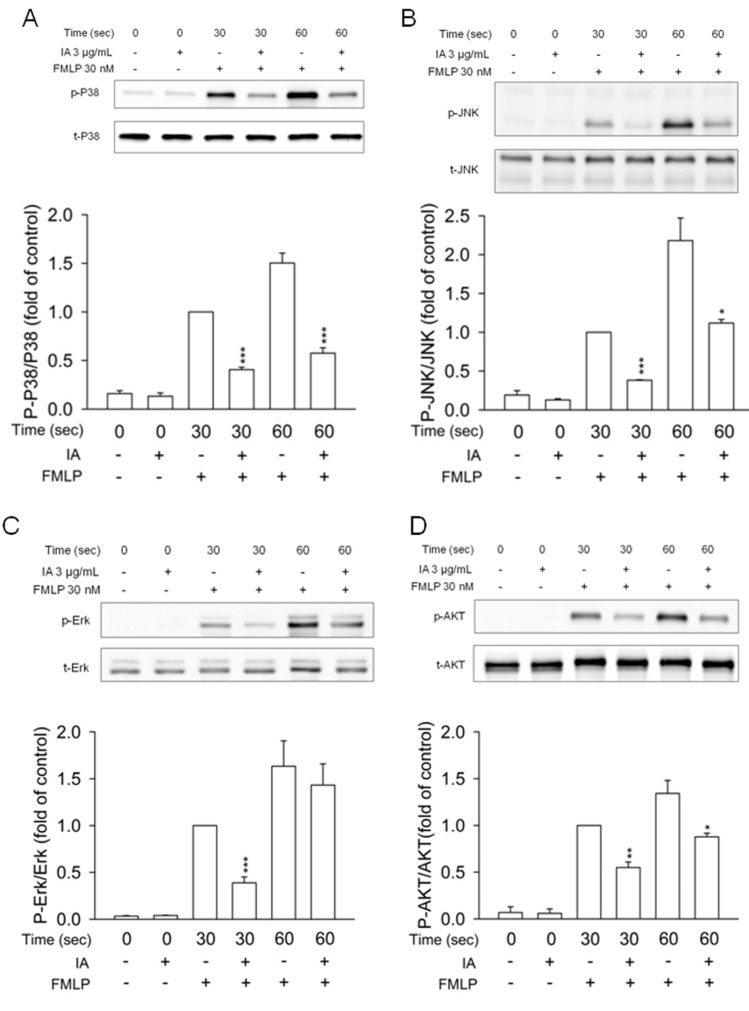
IA inhibits phosphorylation of MAP kinases and AKT in FMLP-activated neutrophils. Neutrophils were treated with IA (3 μg/mL) for 5 min, and then activated with FMLP (30 nM) at 30 and 60 sec. Phosphorylation of MAP kinases and AKT was analyzed with sodium dodecyl sulfate-polyacrylamide gel electrophoresis and immunoblotting. Densitometric analysis of all samples was normalized to the corresponding total protein. All data are summarized as means ± SEM relative to the DMSO group, which activated by FMLP at 30 sec. (n = 3). *p  < 0.05, **p  < 0.01, ***p  < 0.001 *versus* the control group.

It is well known that MAP kinases and phosphatidylinositol 3-kinase/AKT pathways are involved in the downstream signaling of FMLP-stimulated neutrophils [18]. Treatment of human neutrophils with FMLP induced a rapid and significant increase in phosphorylation of p38 MAP kinase, JNK, Erk, and AKT. IA (3 μg/mL) caused a significant reduction of the phosphorylation of MAP kinases and AKT in FMLP-induced neutrophils (Figure 5). These results suggest that IA inhibits the activation of MAP kinases and AKT in FMLP-activated neutrophils.

### 2.6. IA Inhibits the Binding of N-Formyl-Nle-Leu-Phe-Nle-Tyr-Lys-fluorescein (FNLFNYK) in Human Neutrophils

**Figure 6:**
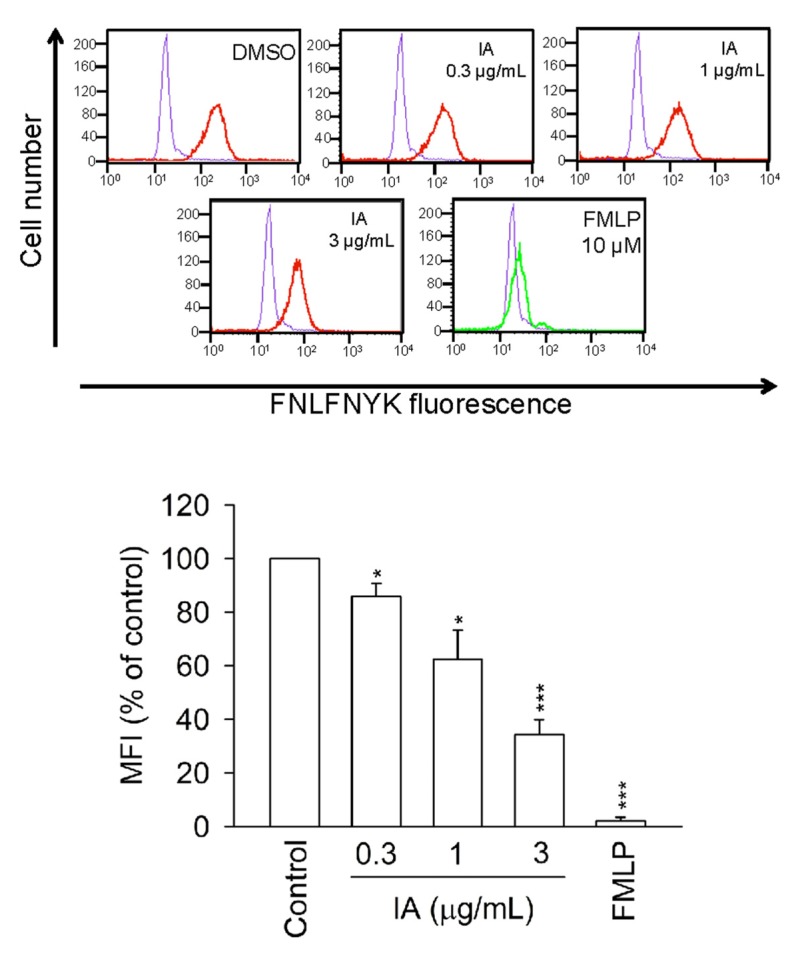
IA inhibits the binding of FNLFNYK in human neutrophils. Neutrophils were incubated with IA or FMLP (10 μM) for 5 min and labeled with the FPR1-specific ligand *FNLFNYK*(4 nM). The purple line meant DMSO alone in the absence of *FNLFNYK*. The red and green line meant testing drugs and FMLP in the presence of FNLFNYK, respectively. The mean fluorescence intensity (MFI) is expressed as the mean ± SEM relative to the control group (100%). (n = 3). * *p* < 0.05, *** *p* < 0.001 *versus* the control group.

To examine whether IA has a binding affinity for FPR1, the binding of FNLFNYK, an FMLP fluorescence analog, to the surface of neutrophils was monitored by flow cytometry. Figure 6 shows that FMLP (10 μM) completely inhibited the binding of FNLFNYK (4 nM) to neutrophils. Compared with the control group, IA significantly and dose-dependently inhibited the binding of FNLFNYK to the FMLP receptor (Figure 6). These results indicate that IA binds to FPR1 in human neutrophils.

### 2.7. IA Inhibits the Binding of FNLFNYK in HEK293 Cells Transfected with FPR1

**Figure 7:**
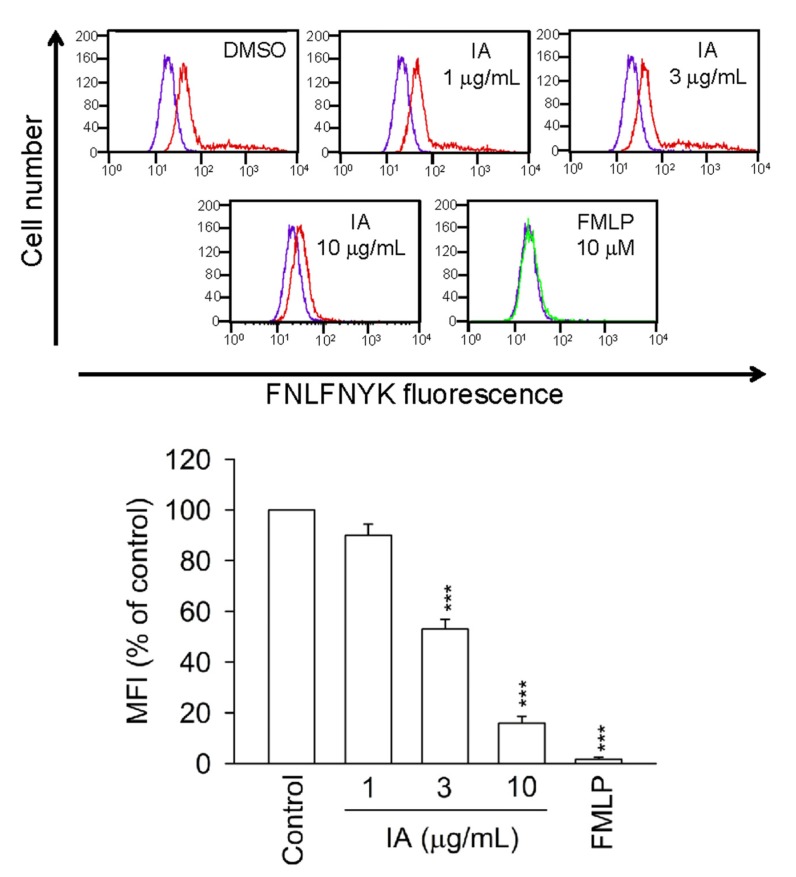
IA inhibits the binding of FNLFNYK in HEK293 cells transfected with FPR1. The tansfected HEK293 cells were incubated with IA or FMLP (10 μM) for 5 min and labeled with the FPR1-specific ligand FNLFNYK (4 nM). The purple line meant DMSO alone in the absence of *FNLFNYK*. The red and green line meant testing drugs and FMLP in the presence of FNLFNYK, respectively. The mean fluorescence intensity (MFI) is expressed as the mean ± SEM relative to the control group (100%). (n = 3). *** *p * < 0.001 *versus* the control group.

To further examine whether IA blocks FPR1, the binding of FNLFNYK on HEK293 cells transfected with FPR1 was monitored by flow cytometry. FMLP (10 μM) blocked the binding of FNLFNYK on HEK293 cells transfected with FPR1 and IA also attenuated the fluorescence on HEK293 cells by blocking FPR1 (Figure 7). These results indicate that IA binds to FPR1 in HEK293 cells transfected with FPR1.

### 2.8. The High Performance Liquid Chromatography (HPLC) Method Used for Identification of the Composition of IA

**Figure 8:**
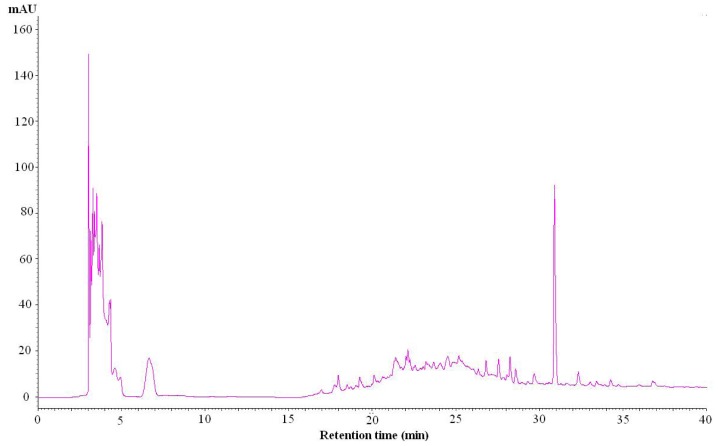
The HPLC fingerprint of IA. The HPLC fingerprint of IAshowed a group of peaks at high-polar fraction (2.5–7.5 min RT) and a significant peak at low-polar fraction (30.9 min RT).

Through a series of experiments, we found the acetonitrile/water elution solvent was a good mobile phase. A photodiode array detector was used to determine the optimized conditions for the IA extract in the chromatogram. In a full-scan experiment, the detector wavelength at 260 nm showed better separation than other wavelengths. The HPLC fingerprint (Figure 8) showed a group of peaks at polar fraction (2.5–7.5 min RT, elution solvent: 2% CH_3_CN) and a significant peak at non-polar fraction (30.9 min RT, elution solvent: 100% CH_3_CN), which was specific enough to be used for the identification of IA.

## 3. Experimental

### 3.1. Reagents

Fluo-3/AM and FNLFNYK were obtained from Molecular Probes (Eugene, OR, USA). MMK-1 was obtained from Tocris Bioscience (Ellisville, MO, USA). *N*-[2-(*p*-Bromocinnamylamino)ethyl]-5-isoquinolinesulfonamide (H89), methoxysuccinyl-Ala-Ala-Pro-Val-nitroanilide was purchased from Calbiochem (La Jolla, CA, USA). Antibodies directed against phosphor-Erk1/2, Erk1/2, phosphor-JNK, JNK, phosphor-AKT (ser-473), and AKT (pan) were from Cell Signaling (Beverly, MA, USA). Antibodies against phosphor-p38 and p38 MAP kinase were obtained from Santa Cruz Biotechnology (Santa Cruz, CA, USA). 2-(4-Iodophenyl)-3-(4-nitrophenyl)-5-(2,4-disulfophenyl)-2*H*-tetrazolium monosodium salt (WST-1) was obtained from Dojindo Laboratories (Kumamoto, Japan). All other pharmacologic agents were purchased from Sigma-Aldrich (St. Louis, MO, USA).

### 3.2. Isolation of Human Neutrophils

Blood were collected from healthy volunteers via venipuncture according to the standard protocol approved by the local institutional review board. Neutrophils were isolated from peripheral blood according to the method of dextran sedimentation, followed by centrifugation in a Ficoll Hypaque gradient and the hypotonic lysis of the erythrocytes [19]. The purified neutrophils contained > 98% viable cells, as determined by Trypan blue exclusion, and were suspended in calcium-free Hank's balanced salt solution (HBSS) at 4 °C before used.

### 3.3. Expression of FPR1 in Human Embryonic Kidney (HEK293) Cells

HEK293 cells were maintained in DMEM supplemented with 10% FBS, 2 mmol/L glutamine, and antibiotics. According to the manufacturer’s instruction, HEK293 cells were stably transfected with the human FPR1 gene. After transfection, cells were cultured in the medium containing G418 (2 mg/mL). G418-resistant clones were used for further studies [20].

### 3.4. Bacterial Strains, Cultivation Condition, and Crude Extract Preparation

The bacterial strain SLI-07-01 was isolated from marine sediment collected at Siaolanyu Isle of Taiwan. This strain was identified by 16S rDNA analysis as a *Bacillus* sp.. The 16S rDNA sequence of SLI-07-01 was deposited in the NCBI Genbank under accession number KC865053. It was maintained on M1 agar (10 g starch, 4 g yeast extract, 2 g peptone, 0.5 L seawater, 0.5 L dH_2_O, and 15 g agar) at 25 °C in Petri dishes [21]. The cultivation of IA was carried out aerobically in 2 L flasks containing 1,000 mL M1 medium with 50% seawater. Flasks were incubated at 25 °C on a rotatory shaker at 150 rpm. After five days of incubation, the fermented broths were extracted twice with ethyl acetate. The solvent extracts were combined and evaporated to dryness under vacuum. The extracts obtained were weighed and stored at −20 °C. A voucher specimen (SF-SLI-07-01) was deposited in the herbarium of National Museum of Marine Biology & Aquarium. The extracts were then used for activity assays.

### 3.5. Measurement of Superoxide Generation and Elastase Release

The measurement of superoxide generated by the activated neutrophils was dependent on the reduction of ferricytochrome *c.* In addition, Methoxysuccinyl-Ala-Ala-Pro-Val-*p*-nitroanilide was used as the elastase substrate to detect elastase release [15]. Briefly, neutrophils were mixed with ferricytochrome *c* or the elastase substrate at 37 °C, and then treated with testing drugs for 5 min. FMLP (30 nM) or MMK-1 (100 nM) in the pretreatment of cytochalasin B as well as PMA (5 nM) were added to activate neutrophils. The change in absorbance was monitored continuously at 550 and 405 nm, respectively, in a spectrophotometer (U-3010, Hitachi, Tokyo, Japan). Calculation was based on the statement from a previous study [22].

### 3.6. Superoxide and DPPH Scavenging Assay

The superoxide-scavenging effect of testing drugs was determined in a cell-free xanthine/xanthine oxidase system. The assay buffer contained 50 mM Tris (pH 7.4), 0.3 mM WST-1, and 0.02 U/mL xanthine oxidase, and testing drugs. After 0.1 mM xanthine was added to the assay buffer at 30 °C, the absorbance related to the reduction of WST-1 by superoxide was measured at 450 nm. In addition, an ethanol solution of the stable nitrogen-centered free radical, DPPH (100 μM), was incubated with testing drugs, and the absorbance was measured at 517 nm.

### 3.7. Evaluation of LDH Release

The assay of cytotoxicity by using LDH was determined by a commercially available method (Promega, Madison, WI, USA). The calculation was based on LDH activity in the testing drugs expressed as a percentage of the total LDH activity. The total LDH activity was determined with the lysis of neutrophils with 0.1% Triton X-100 at 37 °C.

### 3.8. Receptor Binding Assay

FNLFNYK, a fluorescent analogue of FMLP, was used for receptor binding by the fluorescence-activated cell sorting scan analysis. Neutrophils were incubated with testing drugs at 4 °C and labeled with FNLFNYK for 30 min, followed by analysis with flow cytometry [23,24].

### 3.9. Measurement of Intracellular Calcium Concentration ([Ca^2+^]i)

Neutrophils were labeled with Fluo-3/AM (2 µM) at 37 °C. The cytoplasmic calcium levels were measured in a quartz cuvette with a thermostat undergoing continuous stirring by a Hitachi F-4500 spectrofluorometer. The excitation wavelength was 488 nm and the emission wavelength was 520 nm. After cells were treated with testing drugs, stimulants were added in the presence of 1 mM Ca^2+^ to increase [Ca^2+^]_i_. [Ca^2+^]_i_ was calculated from the fluorescence intensity, as follows: [Ca^2+^]_i_ = Kd × [(F − F_min_)/(F_max_ − F)]; where F is the observed fluorescence intensity, F_max_ and F_min_ were obtained by the addition to the neutrophils of 0.05% Triton X-100 and 20 mM EGTA, respectively, and Kd was taken to be 400 nM.

### 3.10. Immunoblotting Analysis

Neutrophils were incubated with testing drugs and stimulated with FMLP (30 nM) at 30 and 60 sec, followed by mixing with sample buffer for 15 min at 100 °C. The reaction was terminated by placing the cells on ice. After centrifugation at 14,000 × *g* for 20 minutes at 4 °C and removal of the supernatant, whole-cell lysates were yielded. The lysates were used for Western blotting analysis. Sodium dodecyl sulfate-polyacrylamide gel electrophoresis with 12% polyacrylamide gels was used to separate the proteins. The samples were then blotted onto nitrocellulose membranes. Immunoblotting was performed with the indicated antibodies and horseradish peroxidase (HRP)-conjugated secondary anti-rabbit antibodies (Cell Signaling Technology, Beverly, MA, USA). The immunoreactive bands were visualized by an enhanced chemiluminescence system (Amersham Biosciences, Piscataway, NJ, USA). The intensities of the reactive bands were analyzed using UVP Biospectrum (UVP, LLC Upland, CA, USA).

### 3.11. HPLC Fingerprint of IA

The HPLC fingerprint of IA was conducted on a Hitachi HPLC system (L-2000 series, Tokyo, Japan). The concentration of extract was 4 mg/mL. The separation was performed on a reverse-phase column (Cosmosil 5C18-AR-II, 5ìm, 25 cm × 4.6 mm I.D.) which was eluted at a flow rate of 1.0 mL/min with a mix solvent of A-B (A = CH_3_CN, B = H_2_O) varying as follows: 0–10 min, 2% A, 98% B; 10–15 min, 2–20% A, 98–80% B; 20–30 min, 20–100% A, 80–0% B; 30–40 min, 100% A, 0% B. The injection volume was 20 μL, and the UV detection wavelength was set at 260 nm.

### 3.12. Statistics Analysis

All experiments were performed at least 3 times and the results are expressed as means ± standard errors of the means (SEM). The statistical analyses were based on ANOVA analysis or Student’s *t*-test, and all calculations were performed with SigmaPlot (Systat Software, San Jose, CA, USA). A value of *p* < 0.05 was considered statistically significant.

## 4. Conclusions

In the present study, we showed that IA, an extract of bioactive secondary metabolites from a marine *Bacillus* sp., exerted anti-inflammatory effects. These results demonstrated that IA inhibited respiratory burst and degranulation specifically in FMLP-activated neutrophils. IA significantly attenuated the downstream signaling pathways of FPR1, including the Ca^2+^, MAP kinases, and AKT pathways. Furthermore, IA inhibited the binding of the FMLP analog to FPR1. Taken together, IA may act as an inhibitor of FPR1. Activation of neutrophils by FPR1 has been proven to be involved in the progress of inflammatory diseases [25]. Therefore, FPR1 has therapeutic potential as a pharmacological target to develop new anti-inflammatory drugs and in conclusion, we show that IA may be a potentially new therapeutic agent against neutrophilic inflammatory diseases.
